# Do coping strategies mediate the effects of childhood adversities and traumata on clinical high-risk of psychosis, depression, and social phobia? A cross-sectional study on patients of an early detection service

**DOI:** 10.1186/s12888-024-06435-2

**Published:** 2025-01-07

**Authors:** Zhixiong Chang, Naweed Osman, Carolin Martha Doll, Theresa Katharina Lichtenstein, Marlene Rosen, Eva Meisenzahl, Hanna Kadel, Joseph Kambeitz, Kai Vogeley, Frauke Schultze-Lutter

**Affiliations:** 1 Department of Psychiatry and Psychotherapy, Medical Faculty, Heinrich-Heine-University, Bergische Landstraße 2, Düsseldorf, 40629 Germany; 2 Department of Psychiatry and Psychotherapy, Faculty of Medicine, University Hospital Cologne, University of Cologne, Cologne, Germany; 3 Research Center Jülich, Institute of Neuroscience and Medicine – Cognitive Neuroscience (INM3), Jülich, Germany; 4 Department of Psychology, Faculty of Psychology, Airlangga University, Surabaya, Indonesia; 5 University Hospital of Child and Adolescent Psychiatry and Psychotherapy, University of Bern, Bern, Switzerland

**Keywords:** Coping, Childhood adversity and trauma, Clinical high-risk, Depression, Social phobia, Mediation

## Abstract

**Background:**

Childhood adversities and traumata (CAT) increase the risk for various mental disorders, including the clinical high-risk of psychosis (CHR-P) state and its main comorbidities, i.e., depression, and social phobia. However, these relations are likely mediated by personal coping behaviors. This cross-sectional study investigates the relationships between the main CAT domains, coping, CHR-P, depression, and social phobia.

**Methods:**

Using path analyses, we analyzed data of 736 patients (mean age 24 years, 67% male) who presented at an early detection service between 2002 and 2013, answered questionnaires on CAT, coping, depressiveness, and social phobia, and underwent clinical examination for CHR-P according to the recommendations of the Guidance project of the European Psychiatric Association.

**Results:**

All path models (total sample, males and females) showed good to excellent fit to the data. In all models, higher scores on maladaptive coping mediated the negative effect of emotional abuse on mental health outcomes. Additionally, in the total sample and males, lower scores on adaptive coping mediated the negative effect of emotional abuse and neglect, and physical neglect was associated with lower scores on adaptive coping that, in turn, were linked to depression and social phobia but not CHR-P. Overall, effects of maladaptive coping were higher than those of adaptive coping, although adaptive coping was more diversely associated with CAT. Furthermore, the interrelated depression and social phobia were more widely explained by the models than CHR-P, which was not significantly associated with them.

**Conclusions:**

Our findings underscore the complex interplay of the CAT domains and their relevant mediators with mental health outcomes that likely reflect underlying sex-specific psychological, social, cultural and neurobiological mechanisms. Supporting a broader view on CAT than the traditional focus on sexual abuse, results indicate an important role of emotional abuse that, descriptively, is most strongly mediated by maladaptive coping strategies on mental health outcomes. A detailed understanding of the effects of CAT will in future help to develop a multi-dimensional, holistic and sex-specific approach to the treatment of patients who have experienced CAT.

**Trial registration:**

The study was registered in the German Clinical Trial Register (https://drks.de/) as DRKS00024469 at 02/24/2021.

**Supplementary Information:**

The online version contains supplementary material available at 10.1186/s12888-024-06435-2.

## Background

The burden of mental disorders in terms of both health and economic losses is immense and increasing, thus calling for preventive actions [1, 2]. To the aim of developing preventive interventions, understanding the determinants and multifactorial, mostly unspecific causes of mental disorders is crucial [2, 3]. Of these, childhood adversities and traumata (CAT) are transdiagnostic risk factors for poor mental health [4, 5], including depression [6], social phobia [7], and psychosis [8], with a high mean prevalence rate of CAT of 86.8% in patients at clinical high-risk of psychosis (CHR-P) [8].

Of the five core domains of CAT, i.e., emotional neglect and abuse, physical neglect and abuse, and sexual abuse, all but sexual abuse were strongly interrelated [9]. A recent path analysis controlling for these interrelations reported that only physical abuse and emotional neglect were associated with adult mental disorders [9]. Thereby, physical abuse was related to depressive, manic, psychotic and anxiety disorders; and emotional neglect was associated with depressive, anxiety and substance use disorders. Furthermore, depressive and anxiety disorders were strongly correlated to each other, while their correlations with psychotic disorders were only weak [9]. These associations may be stronger in early stages of psychoses, as depressive and anxiety disorders, especially social phobia, were frequently reported for CHR-P patients [10] of whom approximately 30% will develop a psychosis within three years [11, 12]. Since the presence of a mental disorder may also increase the risk of developing further mental symptoms, it was assumed that the presence of depression and social phobia may also increase the risk of developing CHR [13].

Among others, the relationship between mental disorders and CAT is mediated by coping behaviors, whereby both positive effects of adaptive coping strategies [14, 15] and negative effects of maladaptive coping strategies [15–19] on various mental health outcomes were reported. Adaptive coping is an essential part of the concept of resilience [20] that describes the ability to adapt one’s own coping behavior to difficult situations and to successfully overcome adversity or trauma [21]. Thus, coping is generally considered an important therapeutic target to ameliorate the negative effects of CAT on mental health and an important target for prevention research [22]. Yet, to date, studies using comprehensive models have not explored the mediating role of coping strategies in the relationship between CAT and CHR-P stages, considering their main comorbidities as outcomes.

In this cross-sectional study, we therefore used path modeling to analyze, if adaptive and maladaptive coping strategies mediate the overall association of CAT (emotional neglect and abuse, physical neglect and abuse, and sexual abuse) with depression, social phobia, and CHR-P in help-seeking patients of an early detection center for psychosis.

## Methods

### Sample and study design

This cross-sectional study was based on data of 2395 patients who sought help for mental problems at an early detection service between 2002 and 2013 [23]. Of this cohort, patients were excluded who had (1) a current or lifetime diagnosis of a psychotic disorder (non-affective, affective, substance-induced) according to DSM-IV or ICD-10 at first contact with the early detection service (*n* = 373; 15.6%); (2) a known diseases of the central nervous system or known somatic diseases with central nervous effects (*n* = 5, 0.2%); (3) clinical or test evidence of IQ < 70 (*n* = 4; 0.2%); (4) insufficient data from the initial survey, in particular lack of information on CHR-P status and/or exclusion of psychosis, for example due to premature termination of the assessment and/or poor language comprehension (*n* = 49, 2.1%); and (5) refusal to use their pseudonymized baseline data in scientific studies (*n* = 111, 4.6%). Of the remaining 1853 eligible patients (77.4%), 736 patients (39.7%) aged 15–52 years had returned the relevant questionnaires on CAT, coping, depression, and social phobia (see ‘Assessments’). They formed the sample of the present analyses.

All subjects (and their legal guardians) had given informed consent to the use of their pseudonymized data in scientific studies. All procedures have been in accordance with the ethical standards of the relevant national and institutional committees on human experimentation and with the Declaration of Helsinki [24]. Ethical approval was granted by the Ethical Committee of the Medical Faculty of the University of Cologne (Reference No. 19-1618_1).

### Assessments

CAT was assessed by the Trauma and Distress Scale (TADS) [25] which is a reliable and valid instrument for assessing retrospectively reported CAT [26]. It includes five core domains: emotional and physical neglect, as well as emotional, physical and sexual abuse. All domains but physical neglect (four items) are based on five items that are rated in a five-point Likert format ranging from 0 = “never” to 4 = “almost always” [26]. Sum scores of the domains were used in the analyses.

Coping behaviors were evaluated by the German Stress-Coping-Questionnaire (SVF-120) [27, 28], which assesses persons’ habitual use of different coping strategies under stress. Each of the 120 items is rated on a 5-point Likert scale ranging from “not at all” to “in any case”. The SVF allows for the calculation of a summary score of positive or adaptive coping strategies, and negative or maladaptive coping strategies from 16 primary scales. The primary scales minimization, trivialization, distraction, situation control, reaction control, positive self-instructions, guilt defense, substitute gratification, self-affirmation, and relaxation are regarded as adaptive strategies. The primary scales social withdrawal, flight, rumination, resignation, self-blame, and self-pity are summarized as maladaptive strategies. The mean sum scores of adaptive and maladaptive strategies entered the analyses.

Depression was assessed by German version of the Beck Depression Inventory (BDI) [29] and, since 2008, by its revision (BDI-II) [30], which are self-report instruments of depression in the past 2 weeks and consist of 21 items. The BDI and BDI-II are reliable instruments to assess the severity of depressive symptoms and are widely used among both adults and adolescents [31]. Because of the differences between the two versions in the content of four items and their different norms (BDI: > 18 = at least moderate depression, BDI-II: > 19 = at least moderate depression), scores were dichotomized according to the thresholds for rating at least a moderate, likely clinically relevant depression provided in the respective manuals, with higher score being rated as depression and equal or lower scores as no depression. This binary variable entered the analyses.

Social phobia was assessed by the German version of the Social Phobia and Anxiety Inventory (SPAI) [32, 33], which is a reliable, valid and commonly used self-report instrument to measure social anxiety in both females and males [34]. In line with the data preparation of the BDI, scores were dichotomized according to the provided threshold into social phobia present (> 2.60) or absent (≤ 2.60). This binary variable entered the analyses.

CHR-P was defined according to the recommendations given in the Guidance project of the European Psychiatric Association [12] by the presence of symptomatic ultra-high risk criteria defined by attenuated and/or transient psychotic symptoms, and/or the basic symptom criterion “Cognitive Disturbances”. The ultra-high risk criteria were assessed by the Structured Interview for Psychosis-Risk Syndromes (SIPS) [35]. “Cognitive Disturbances” was assessed by the Schizophrenia Proneness Instrument, Adult version [36]. Both semi-structured clinical interview assessments of CHR-P were reported to have good interrater reliability for trained raters [37], such as the clinicians working in the early detection service from that the sample was recruited.

### Statistical analyses

First, categorical data were compared by χ^2^-tests, non-normally distributed ratio and ordinal data by Mann–Whitney U tests. Second, the sum scores of the five TADS domains, and the two SVF subscales (adaptive and maladaptive coping) were included as continuous predictors in the path analysis, while CHR-P status, depression and social phobia were entered as dichotomous outcome variables. The paths from CAT via coping mechanisms to mental health disorders were modelled by including all possible direct associations between CAT and coping, and coping and clinical outcomes, respectively. Missing items were accounted for by using the estimator full information maximum likelihood [38]. The comparative fit index (CFI ≥ 0.95), the root-mean-square error of approximation (RMSEA ≤ 0.06) and the standardized root mean square residual (SRMR ≤ 0.08) were used to evaluate model fits [39, 40]. Usefulness of the χ^2^-statistic as a fit indicator is limited by its sensitivity to sample size and its tendency to reject models in large samples like ours [39]. Therefore, we followed the “2-index presentation strategy” by Hu and Bentler [41] that assumes that a path model is well fitting, if RMSEA and its 90% confidence intervals (CIs) are ≤ 0.06, and SRMR ≤ 0.08. Additionally, sensitivity models were calculated separately for female and male patients. Furthermore, to test for the effect of dichotomizing BDI and SPAI, a step that eliminates information and might reduce reliability of the measures, we also rerun the models with continuous sum scores of BDI and SPAI, thereby mixing both BDI versions.

After calculation of the path model, mediating effects were tested in separate small models containing three variables each for the paths indicating mediation using the criteria according to Zhao et al. [42] as well as Rucker et al. [43]. The statistical analyses were conducted in SPSS 27.0 and the R language for statistical computing using the packages “lavaan” [44] and “psych” [45]. Throughout, we considered a level of significance of α < 0.05.

## Results

### Sample characteristics

Of the 736 patients, 246 (33.4%) were females and 490 (66.6%) were males (Table 1). Their median age was 24 years and did not differ between sexes. Altogether 54% had received any clinical diagnoses, 31% met criteria of a CHR-P state and 49% had a positive family history of any mental disorders, with no differences between sexes. Social phobia according to the SPAI (56%) did not differ between sexes, too, while depression according to the BDI was more frequent in females (78%) than males (66%). Sexual abuse was rare and slightly more frequent in females, and also both emotional abuse and neglect was more pronounced in females. Only physical abuse was more severe in males; physical neglect did not differ between sexes (Table 1).

**Table 1:** Sociodemographic and clinical characteristics of the sample

	**Males (*n* = 490, 66.6%)**	**Females (*n* = 246, 33.4%)**	**Total (*N* = 736, 100%)**	**Statistics; effect size**^**a**^
**age**, median (mean ± SD)	23 (24.60 ± 5.51)	24 (25.06 ± 5.99)	23.96 (24.75 ± 5.68)	U = 57,581.0, *p* = 0.322; *r* = -0.017
**nationality,** % German,	93.1%	92.3%	92.8%	χ^2^(1) = 0.056, *p* = 0.756; V = 0.015
**highest education**, %				χ^2^(5) = 6.334, *p* = 0.260; V = 0.095
ISCED 2	33.3%	26.0%	30.9%	
ISCED 3	50.6%	54.5%	51.9%	
ISCED 4	1.1%	2.6%	1.6%	
ISCED 5	1.7%	1.7%	1.7%	
ISCED 6	4.9%	4.7%	4.8%	
ISCED 7	8.3%	10.6%	9.1%	
**marital status,** %				χ^2^(4) = 21.701, *p* < 0.001; V = 0.172
single	74.3%	63.0%	70.5%	
divorced/widowed	1.6%	0.0%	1.1%	
married	2.4%	7.3%	4.1%	
steady relationship	21.2%	28.5%	23.6%	
**psychosocial functioning**^b^**,** median (mean ± SD)	55 (56.43 ± 14.25)	59.5 (59.22 ± 15.16)	55 (57.35 ± 14.60)	U = 31,993, *p* = 0.038; *r* = -0.074
**mental disorder**^c^**,** % any one present	53.1%	54.9%	53.7%	χ^2^(1) = 0.150, *p* = 0.695; V = 0.017
depressive disorder	27.6%	27.2%	27.4%	χ^2^(1) < 0.001, *p* = 0.997; V = 0.003
bipolar disorder	2.4%	2.0%	2.3%	χ^2^(1) = 0.009, *p* = 0.801; V = 0.013
anxiety disorder	12.7%	13.8%	13.0%	χ^2^(1) = 0.107, *p* = 0.645; V = 0.016
obsessive compulsive disorder	6.7%	4.5%	6.0%	χ^2^(1) = 1.117, *p* = 0.252; V = 0.045
adjustment disorder	7.6%	9.3%	8.2%	χ^2^(1) = 0.488, *p* = 0.395; V = 0.031
post-traumatic stress disorder	0.2%	0.4%	0.3%	χ^2^(1) < 0.001, *p* = 1.000; V = 0.018
eating disorder	0.2%	3.3%	1.2%	χ^2^(1) = 10.199, *p* < 0.001; V = 0.131
somatization disorder	2.4%	2.4%	2.4%	χ^2^(1) < 0.001, *p* = 1.000; V < 0.001
other mental disorder	16.5%	14.2%	15.8%	χ^2^(1) = 0.492, *p* = 0.454; V = 0.030
**alcohol misuse,** % present	4.5%	0.8%	3.3%	χ^2^(1) = 5.901, *p* = 0.007; V = 0.098
**illicit drug misuse,** % present	8.6%	4.5%	7.2%	χ^2^(1) = 3.530, *p* = 0.049; V = 0.075
**psychopharmacological medication,** % current intake of any	33.3%	28.9%	31.8%	χ^2^(1) = 1.309, *p* = 0.240; V = 0.045
**mental disorder in family,** % any present	48.8%	47.6%	48.4%	χ^2^(1) = 0.054, *p* = 0.815; V = 0.011
**CHR-P criteria,** % present	29.0%	34.4%	30.8%	χ^2^(1) = 2.036, *p* = 0.149; V = 0.056
**social phobia:** % present acc. to SPAI	55.7%	56.0%	55.8%	χ^2^(1) < 0.001, *p* = 1.000,V = 0.002
**depression,** % present acc. to BDI	66.1%	77.6%	69.8%	χ^2^(1) = 7.707, *p* = 0.004,V = 0.117
**adaptive coping**, median (mean ± SD)	10.9 (10.75 ± 2.94)	10.3 (10.19 ± 3.09)	10.7 (10.56 ± 2.99)	U = 54,658, *p* = 0.024; *r* = -0.077
**maladaptive coping**, median (mean ± SD)	12.7 (12.67 ± 4.31)	14.0 (13.69 ± 3.95)	13.2 (13.01 ± 4.22)	U = 42,917, *p* = 0.004; *r* = -0.102
**physical abuse**, median (mean ± SD)	5.0 (5.29 ± 1.81)	4.0 (5.02 ± 1.9)	5.0 (5.2 ± 1.84)	U = 68,839, *p* < 0.001; *r* = -0.115
**physical neglect**, median (mean ± SD)	4.0 (4.68 ± 3.14)	4.0 (4.9 ± 3.48)	4.0 (4.75 ± 3.26)	U = 58,744, *p* = 0.573; *r* = 0.007
**sexual abuse**, median (mean ± SD)	0.0 (0.84 ± 2.50)	0.0 (1.82 ± 3.91)	0.0 (1.16 ± 3.07)	U = 50,227, *p* < 0.001; *r* = -0.171
**emotional abuse**, median (mean ± SD)	4.0 (4.85 ± 4.23)	5.0 (5.89 ± 4.94)	4.0 (5.2 ± 4.50)	U = 53,689, *p* = 0.015; *r* = -0.080
**emotional neglect**, median (mean ± SD)	7.0 (7.79 ± 4.4)	9.0 (8.84 ± 5.13)	8.0 (8.14 ± 4.68)	U = 53,051, *p* = 0.008; *r* = -0.090

^a^Effect sizes: V: Cramer’s V for categorical variables, r: Rosenthal’s r for ordinal variables and non-normally distributed parametric variables;

^b^according to the Social and Occupational Functioning Assessment Scale [46] with 1 = ’persistent hygiene problems’ to 100 = ’superior functioning’

^c^clinical diagnoses

*CHR-P* clinical high-risk of psychosis

*ISCED* International Standard Classification of Education (2011)

*SD* standard deviation

*SPAI* Social Phobia and Anxiety Inventory

*BDI* Beck Depression Inventor

### Associations between CAT, coping, and mental health outcomes

**Fig. 1:**
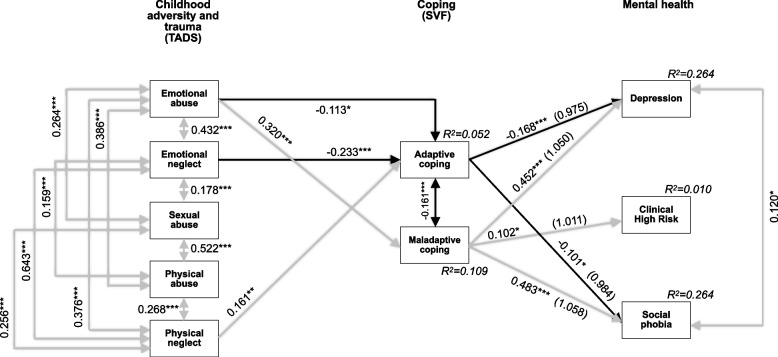
Final model of associations between traumatic experience, coping and mental health with standardized significant path coefficients (*N* = 736). Model fit indices: χ2(15) = 28.605 with *p* = 0.018, CFI = 0.990, SRMR = 0.029, RMSEA = 0.035 (90%CI = 0.014–0.054). Power = 0.948. *p ≤ 0.05; **p ≤ 0.01; ***p ≤ 0.001. *TADS* Trauma and Distress Scale. German Stress-Coping-Questionnaires (SVF). Explained variance (R^2^) for each endogenous variable in italics. Odds ratios in brackets for the endogenous variables,,Adaptive coping” and,,Maladaptive coping”. Solid lines indicate significant paths (*p* ≤ 0.05), grey indicates positive associations, black indicates negative associations

Our final cross-sectional model (Fig. 1), combining males and females, showed excellent fit and power (0.948). At significance level *p* ≤ 0.001, all endogenous CAT domains of the TADS correlated positively with each other. Emotional abuse and neglect were both negatively associated with adaptive coping, and physical neglect was positively associated with adaptive coping. Additionally, emotional abuse was positively associated with maladaptive coping. No significant associations between sexual abuse or physical abuse, and coping were found. Adaptive coping was negatively associated with depression and social phobia but not CHR-P; and maladaptive coping showed positive associations with depression, CHR-P and social phobia. Adaptive coping and maladaptive coping were negatively associated with each other. While depression and social phobia were positively associated with each other, CHR-P did not show any significant associations with either of these (Fig. 1).

### Sensitivity models for sex

**Fig. 2:**
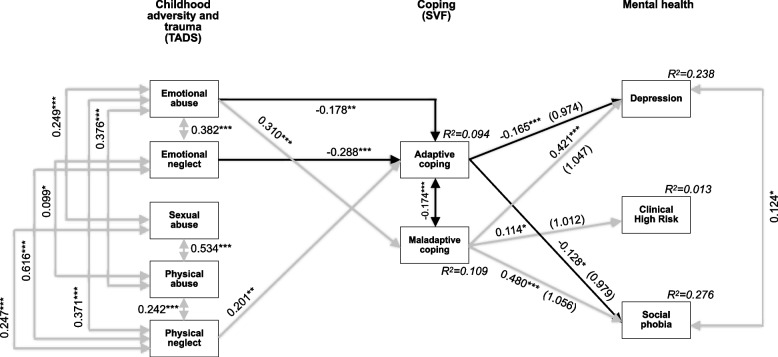
Model of associations between traumatic experience, coping and mental health with standardized significant path coefficients for male patients (*N* = 490). Model fit indices: χ2(15) = 23.412 with *p* = 0.076, CFI = 0.991, SRMR = 0.032, RMSEA = 0.034 (90%CI = 0.000–0.059). Power = 0.787. *p ≤ 0.05; **p ≤ 0.01; ***p ≤ 0.001. *TADS* Trauma and Distress Scale. German Stress-Coping-Questionnaires (SVF). Explained variance (R^2^) for each endogenous variable in italics. Odds ratios in brackets for the endogenous variables,,Adaptive coping” and,,Maladaptive coping”. Solid lines indicate significant paths (*p* ≤ 0.05), grey indicates positive associations, black indicates negative associations

The cross-sectional model for male patients who make two thirds of the sample (Fig. 2) equaled the model on the total sample in all but the non-significant association between sexual abuse and emotional neglect. Yet, while model fit was still excellent, the power was decreased to 0.787.

**Fig. 3:**
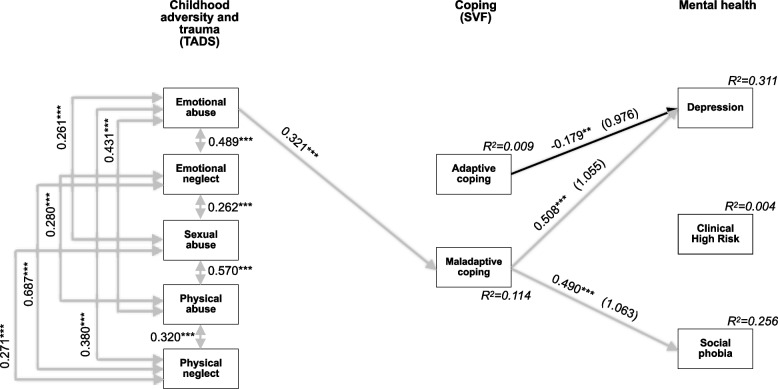
Model of associations between traumatic experience, coping and mental health with standardized significant path coefficients for female patients (*N* = 246). Model fit indices: χ2(15) = 14.835 with *p* = 0.463, CFI = 1.000, SRMR = 0.031, RMSEA = 0.000 (90%CI = 0.000–0.060). Power = 0.420. *p ≤ 0.05; **p ≤ 0.01; ***p ≤ 0.001. *TADS* Trauma and Distress Scale. German Stress-Coping-Questionnaires (SVF). Explained variance (R^2^) for each endogenous variable in italics. Odds ratios in brackets for the endogenous variables,,Adaptive coping” and,,Maladaptive coping”. Solid lines indicate significant paths (*p* ≤ 0.05), grey indicates positive associations, black indicates negative associations

The cross-sectional model for the smaller sample of female patients (Fig. 3) had excellent fit but poor power (0.420) and greatly differed from the total sample and male models. While all CAT domains were again positively associated, coping strategies and mental health outcomes, respectively, showed no significant intra-dimensional associations. The only significant direct path between CAT domains and coping was between emotional abuse and maladaptive coping, which was again negatively associated with depression and social phobia. Furthermore, the negative association between adaptive coping and depression showed as well (Fig. 3).

### Mediation analyses

In the path model on the whole sample, nine mediations were indicated and, thus, tested for a mediation effect (Table 2). Six mediation analyses revealed significant direct, indirect and total effects supporting rather small complementary mediation effects of coping according to Zhao et al. [42] in the following paths: from emotional abuse and emotional neglect, respectively, via adaptive coping to depression and social phobia, as well as from emotional abuse via maladaptive coping to depression and social phobia. The mediation analysis of the path from emotional abuse via maladaptive coping to CHR-P revealed merely a significant small indirect but no significant direct or total effect supporting a small indirect-only mediation effect of maladaptive coping according to Zhao et al. [42].

**Table 2:** Standardized path coefficients of mediation analyses in total sample (*N* = 736)

**Independent variable**	**Mediator variable**	**Dependent variable**	**Direct effect**^a^	**Indirect effect**^**b**^	**Total effect**^**c**^
emotional abuse	adaptive coping	depression	0.238***	0.032**	0.270***
emotional abuse	adaptive coping	social phobia	0.222***	0.027**	0.248***
emotional neglect	adaptive coping	depression	0.160***	0.039***	0.199***
emotional neglect	adaptive coping	social phobia	0.226***	0.029**	0.255***
physical neglect	adaptive coping	depression	0.100*	0.006	0.106**
physical neglect	adaptive coping	social phobia	0.126**	0.005	0.131**
emotional abuse	maladaptive coping	depression	0.116**	0.142***	0.259***
emotional abuse	maladaptive coping	clinical high-risk	-0.001	0.033*	0.032
emotional abuse	maladaptive coping	social phobia	0.085*	0.150***	0.235***

^*^p ≤ 0.05 **p ≤ 0.01 ***p ≤ 0.001

^a^Direct effect = Effect of independent variable on dependent variable (c)

^b^Indirect effect = Product of effects of independent variable on mediator variable (a) and mediator variable on dependent variable (b)

^c^Total effect = c + (a*b)

Only non-significant indirect effects of negligible size (< 0.01) were found for adaptive coping in the paths from physical neglect to depression and social phobia, respectively; for these paths, only direct and total effects became significant (Table 2).

The sensitivity model of the male patients indicated the same nine mediations as the model of the whole sample, and revealed similar significant indirect, direct and total effects of similar size (Table 3). In the sensitivity model of the female patients, only two mediations were indicated and tested (Table 4). The results revealed significant direct, indirect and total effects only for the mediation analysis of emotional abuse via maladaptive coping to depression supporting a rather large complementary mediation effect of maladaptive coping according to Zhao et al. [42]. The mediation analysis of emotional abuse via maladaptive coping to social phobia, however, revealed merely a significant indirect but no significant direct effect supporting a rather large indirect-only mediation effect of maladaptive coping according to Zhao et al. [42]. Descriptively, these mediation effects were the highest of all mediation effects tested in the three models.

**Table 3:** Standardized path coefficients of mediation analyses in male patients (*N* = 490)

**Independent variable**	**Mediator variable**	**Dependent variable**	**Direct effect**^a^	**Indirect effect**^**b**^	**Total effect**^**c**^
emotional abuse	adaptive coping	depression	0.192***	0.045**	0.237***
emotional abuse	adaptive coping	social phobia	0.251***	0.044**	0.295***
emotional neglect	adaptive coping	depression	0.161***	0.049***	0.210***
emotional neglect	adaptive coping	social phobia	0.246***	0.046**	0.292***
physical neglect	adaptive coping	depression	0.097*	0.010	0.107*
physical neglect	adaptive coping	social phobia	0.158**	0.012	0.170**
emotional abuse	maladaptive coping	depression	0.095*	0.129***	0.225***
emotional abuse	maladaptive coping	clinical high-risk	0.022	0.032*	0.053
emotional abuse	maladaptive coping	social phobia	0.130*	0.144***	0.274***

^*^p ≤ 0.05 **p ≤ 0.01 ***p ≤ 0.001

^a^^bc^See Table 2

**Table 4:** Standardized path coefficients of mediation analyses in female patients (*N* = 246)

**Independent variable**	**Mediator variable**	**Dependent variable**	**Direct effect**^a^	**Indirect effect**^**b**^	**Total effect**^**c**^
emotional abuse	maladaptive coping	depression	0.140*	0.160***	0.300***
emotional abuse	maladaptive coping	social phobia	0.035	0.154***	0.189**

^*^p ≤ 0.05 **p ≤ 0.01 ***p ≤ 0.001

^abc^See Table 2

### Replication of the model with continuous depression and anxiety scores

Notwithstanding the differences between the two BDI versions, the model remained essentially the same using the raw sum scores of both BDI and SPAI, with descriptively slightly higher standardized path coefficients for the associations of coping with BDI and SPAI scores [see Additional file 1]. Yet, with regard to model fit, this ‘metric’ model performed slightly poorer than the original model (χ2(15) = 80.923 with *p* < 0.001, CFI = 0.961, SRMR = 0.045, RMSEA = 0.077 (90%CI = 0.061–0.094)), with RMSEA not meeting the requirements of the “2-index presentation strategy” [41]. For the nine indicated mediations, the same pattern of significant direct and indirect effects as in the original sample revealed, with descriptively generally slightly higher path coefficients [see Additional file 2]. With regard to the original sex models, these were also replicated when using the continuous BDI and SPAI data; yet, again with poorer model fit [see Additional files 3, 4, 5 and 6].

## Discussion

In this cross-sectional study, we examined the role of adaptive and maladaptive coping behaviors in the association of the five main CAT domains, i.e., emotional neglect and abuse, physical neglect and abuse, and sexual abuse, with a CHR-P state and its two most frequently reported comorbid conditions, i.e., depression and social phobia [10]. Our total sample model, which predominately reflects associations in males, demonstrated a mediating role of both adaptive and maladaptive coping in the association of emotional abuse, and physical and emotional neglect, with poor mental health outcome, in particular with the interrelated depression and social anxiety. A CHR-P state was least explained, and only related to emotional abuse via maladaptive coping in the total sample and males, and, unexpectedly, not significantly associated with depression or social phobia in both sexes. Interestingly, while maladaptive coping was generally positively and adaptive coping negatively associated with CAT, physical neglect was positively associated with adaptive coping in the total and the male sample.

### The mediating role of adaptive and maladaptive coping

Our results support previous, mostly cross-sectional studies [14–19], which reported that coping behaviors would function as mediators between CAT and mental health outcomes. Yet, while earlier studies commonly reported either adaptive [14] or maladaptive coping [15–19] as a mediator between CAT and poor mental health in various population and patient samples, in line with a cross-sectional model on siblings of patients with non-affective psychosis [15], both maladaptive and adaptive coping strategies mediated the impact of CAT on mental health in our sample. In doing so, reflecting the dominance of studies reporting mediation effects of maladaptive coping and results of a structural equation modelling meta-analysis on the association of control and competence beliefs, coping and mental health [47], the association of maladaptive coping with depression and social phobia in our model was about three times higher than the association of adaptive coping, and even exclusive on a CHR-P state. This finding supports the dominant mediation role of maladaptive coping strategies in the association between CAT, especially emotional abuse, and mental health outcome, despite the broader range of associations of adaptive coping strategies with CAT domains.

In agreement with a cross-sectional study of mediators between CAT and suicidality in CHR-P patients [14], the effects of emotional abuse and neglect on depression and social phobia were mediated by lower scores of adaptive coping in our total and the male sample. Additionally, in line with reports on the mediation role of various maladaptive coping strategies in the relation between global measures of CAT and various mental health outcomes [15–19], descriptively, the specific effect of emotional abuse on mental health was even more strongly mediated by higher scores of maladaptive coping in our study. Thereby, maladaptive and adaptive coping were negatively related, albeit to a lesser degree than reported in a structural equation modelling meta-analysis [47].

Thus, preventing or reducing maladaptive coping strategies may have greater impact on mental health than facilitating the development of adaptive coping. Yet, for the cross-sectional nature of our study, and despite the assumed trait character and thus the advance of coping strategies before the occurrence of current mental disorders [48], depressive and anxiety disorder often occur early in life [49, 50] and thus might have shaped coping strategies. Thus, a reciprocal association cannot be ruled out. The CHR-P criteria, however, have been explicitly defined as state criteria of non-trait character, and have been shown to vary significantly more over time than trait-like schizotypy measures [51]. Therefore, more than in case of depression and social anxiety, CHR-P criteria can be assumed to occur after both CAT and the development of coping strategies.

### The non-significant role of sexual abuse

Sexual abuse is the mostly studied childhood adversity [52], and meta-analyses of individual effects of CAT domains [4, 53–55] have reported strong associations between sexual abuse and mental disorders, in particular depressive and anxiety disorders. Yet, these studies did not account for the common co-occurrence of CAT domains [9, 54]. And in a recent path model of direct effects of CAT on mental disorders that accounted for the interrelations of the five CAT domains and of diagnostic main categories, respectively [9], in line with our study, a lack of a significant association of sexual abuse with mental health was reported. This model also found significant interrelations between all five CAT domains, whereby sexual abuse was the least frequently reported and had the lowest interrelations with the other CAT domains [9]. These findings are mostly in line with our findings, except for the strong interrelation between sexual and physical abuse that partly supports the strong association between sexual and other forms of abuse [56].

Because female sex was a predictor of sexual abuse [56], a reason for the lack of a significant role of sexual abuse in our main model might be the low number of females (33.4%) in our study. Yet, in the study of Salokangas et al. [9], females (71.8%) had been in the majority; and in both our and Salokangas’ study, sexual abuse was equally rare and weakly related to female sex. Thus, for the reported dose–response relationship between multiple forms of CAT and mental disorder [57] and its description as the most synergistically reactive CAT domain [58], sexual abuse may mainly add to the overall burden of CAT in those most likely afflicted also by other forms of CAT.

### The pathogenic role of emotional neglect and abuse

As regards the role of non-sexual CAT domains, other than in the model of Salokangas et al. [9] that found only direct effects of emotional neglect and physical abuse on mental disorders, in particular depressive and anxiety disorders; in our sample, also emotional abuse, and physical neglect but not physical abuse showed significant direct effects on depression and social phobia but not CHR-P in the mediation analyses of the total and male sample; in females, only emotional abuse was directly related to depression. Yet, Salokangas’ model [9] had only examined direct effects between CAT and mental disorders, while our model had focused on the mediating role of coping and had not included direct paths between CAT domains and mental health outcomes in the overall model.

Our mediation analyses suggest that, in the total and male sample, most direct effects of CAT domains on both depression and social anxiety have higher absolute values than the related indirect effects via coping. Only in the paths from emotional abuse via maladaptive coping to mental health outcomes, the indirect effects had higher absolute values than the direct ones in all models. Thus, some direct paths between CAT domains and mental disorders reported by Salokangas et al. [9], in particular paths between physical abuse, and depression, social phobia and CHR-P, might have been missed in our study for lack of their relation to coping.

### The resilience enhancing role of physical neglect

An unexpected finding in both the total and the male sample model was the positive association of physical neglect with adaptive coping. Yet, adaptive coping did not act as a mediator between physical neglect, and depression or social phobia so that this positive association should not be interpreted as a mental health enhancing effect of physical neglect but may indicate the possibility of a resilience enhancing effect of physical neglect.

Resilience had been defined, among others, as a “process of overcoming (or thriving or succeeding) difficulties, adversity, or trauma to a point of becoming more successful or functioning even better than before” [p.460, 20]. Accordingly, the inoculation model of resilience assumes that adequate levels of challenges facilitate development of promotional factors, i.e., assets, which include adaptive coping, good stress management and problem-solving skills [20]. Thus, as for traumata in later life that can lead to posttraumatic growth [59], not only were links of CAT with later mental disorder described but also links with increased resilience, in terms of good coping skills, self-efficacy, adaptive locus of control or good sense of coherence, in particular when social support was provided [47, 60–64]. Besides such psychological and environmental factors that may explain or have mediated the positive association of physical neglect and adaptive coping in our model, biological factors might have played a role. Among these are an adequate hypothalamic–pituitary–adrenal axis function with appropriately strong corticosteroid (especially cortisol) elevation in times of stress and a favorable brain network architecture with good efficacy of certain nodes, which include the right amygdala that plays a role in the expression of fear and in the processing of fear-inducing stimuli, and the right subcallosal gyrus that suppresses the responsiveness of the amygdala to fearful cues [60, 65–69]. Future studies should examine the interplay of relevant psychological, environmental and biological factors to better understand the different damaging as well as promoting effects of various CAT.

### The association of CAT, coping and clinical high-risk for psychosis

A recent meta-analysis of the population attributable fraction (PAF) of potentially modifiable risk factors for mental disorders [4] reported the largest global PAFs for CAT and schizophrenia spectrum disorders, and for sexual abuse and depressive disorders. A meta-analysis on environmental risk factors for psychosis in CHR-P samples [70] showed a significant association of emotional abuse and, though less strongly, of physical neglect with a CHR-P state, whereas another meta-analysis on the prevalence of CAT in CHR-P compared to healthy controls [71] found significantly increased rates of emotional and physical abuse but not of sexual abuse, and emotional and physical neglect in CHR-P patients. Thus, emotional abuse was the most consistently reported influential CAT domain in CHR-P. While we found no direct association between this domain and an CHR-P state, it was the only CAT domain with at least an indirect association with CHR-P via higher scores of maladaptive coping in the total sample and the male model but not the female model. Furthermore, in the total sample and male models, CAT and coping explained far less variance of presence of a CHR-P state (1%) compared to the presence of depression and social phobia (around 25% each in the original and around 40% each in the ‘metric’ model). This difference in explained variance was even extended in the female model.

Unexpectedly in light of reports of the frequent co-occurrence of CHR-P states with depression and anxiety disorders, especially social phobia [10], in all our models, a CHR-P state was not significantly linked to these disorders. While this might be related to their definition by self-rating questionnaires that resulted in higher rates than the respective clinical diagnosis, this could also indicate that most of the association of CAT, coping and CHR-P were explained by the associations of CAT and coping with the main comorbidities of a CHR-P state. This explanation would be roughly in line with the path model of Salokangas et al. [9] in that depressive and anxiety disorders showed more significant paths and higher path coefficients linked with CAT, and higher path coefficients between each other in comparison to their associations with psychotic disorders. Thus, the associations of CAT and coping with depression and social phobia may be independent of the descriptively weaker link between CAT, coping and a CHR-P state.

This weak link might also be explained by our definition of a CHR-P state that was not only defined by ultra-high risk criteria (as in most of the CHR-P studies) but also by the basic symptom criterion Cognitive Disturbances. Differential relations of these two approaches with CAT were demonstrated in an earlier study on mediators between CAT and suicidality in CHR-P patients [14]. While both ultra-high risk symptoms and cognitive basic symptoms were linked to CAT, only Cognitive Disturbances mediated the link between CAT and suicidality [14].

Also with respect to (attenuated) psychotic symptoms, differential associations with CAT domains had been reported in both clinical and community samples. A recent meta-analysis of psychological processes involved in the association between psychotic symptoms and CAT [72] reported emotional dysregulation, dissociative and post-traumatic stress disorder symptoms as moderators of the association between CAT and hallucinations, and negative schemata as moderators of the association between CAT and both delusions and paranoia. Obsessive–compulsive phenomena, attachment, and social cognition were associated with CAT but unrelated to psychotic symptoms; the potential role of depressive and anxiety symptoms, of coping, and of specific CAT domains were not examined in this meta-analysis [72]. When CAT domains were considered in a CHR-P sample recruited by ultra-high risk criteria, negative self-schema partially mediated the effect of emotional neglect on CHR-P status in general and paranoia in particular, even after controlling for the effects of previous exposure to cannabis use [73]. Another study on CHR-P patients according to the ultra-high risk criteria found no direct effects of threat (abuse) or deprivation (neglect) exposure on any of the five psychotic symptom domains of the SIPS [74] but a unique link was identified instead between abuse, and delusional thinking and paranoia that was mediated by negative cognitive schemas about others [74]. In ultra-high risk individuals identified by the SIPS in a large Brazilian household survey [75], path analyses showed that physical and emotional neglect were inversely related to grandiosity, physical abuse was related to perceptual aberrations, and physical neglect was related to disorganized communication, while sexual and emotional abuse were unrelated to any attenuated psychotic symptom. In another non-clinical sample examined for attenuated psychotic symptoms with the SIPS, sexual abuse was significantly associated with higher scores of disorganized communication (mean ± SD: 0.60 ± 0.87) and emotional neglect was significantly associated with more severe paranoid ideas (mean ± SD: 1.15 ± 1.26), above and beyond the effect of other co-occurring traumas [76]. Grandiose or other delusional thinking and perceptual aberrations were unrelated to any CAT domain [76]. Yet, scores on SIPS positive items were mostly well below the minimum score to rate attenuated psychotic symptoms (i.e., a score of “3”); consequently, these results mostly reflect associations with more general symptoms such as wariness, hypervigilance and indistinct concerns about safety, or slightly vague, muddled, or overelaborated speech. These studies indicate, on the one hand, differential influences of CAT domains on specific positive symptom and CHR-P states and, on the other hand, a possibly stronger influence of negative schemas, competence and control beliefs in the association between CAT and CHR-P that may even further moderate the effect of coping [14, 47]. Thus, as differences between study findings are likely related to different compositions of CHR-P samples with respect to their defining symptoms and the choice of possible moderators, future large studies on the association of CHR-P states and CAT should include a broader range of possible moderators, including protectors, and differentially examine CAT and symptom domains.

### The influence of sex

Our sensitivity analyses indicated several differences between female and male patients. Consistent with previous reports of female sex predicting depression [77] and of a lack of sex difference in social phobia [78], depression according to the BDI was more frequent in females, while social phobia according to the SPAI did not differ between sexes. In line with a systematic review of sex differences in the prevalence of CAT domains in European samples [52], sexual and emotional abuse was most pronounced in females, while physical abuse was most pronounced in males. Neglect did not differ between sexes in the systematic review that did not distinguish between emotional and physical neglect [52]. This lack of a sex difference also showed for physical neglect in our study, while emotional neglect was more pronounced in females. Coping strategies were also reported to differ between sexes with females generally engaging more in all strategies [79]. In our study, this was only the case for maladaptive coping strategies that were slightly more pronounced in females. Adaptive strategies were slightly more pronounced in males; yet, descriptively, this sex effect was even smaller than the weak one for maladaptive strategies and, thus, almost negligible.

In line with these simple sex differences, the path models of males and females greatly differed. While the male model was equal to the total sample model and showed multiple significant paths linking CAT with other measures directly and indirectly; in female patients, emotional abuse was the only CAT domain associated via maladaptive coping with mental health outcomes, whereby maladaptive coping complementarily mediated the link to depression and fully mediated the association with social phobia. Furthermore, in female patients, coping was not associated with CHR-P, and adaptive coping was not associated with any CAT domain. While the lower number of significant paths in females might be due to the smaller sample size and lower power compared to the male sample, the missing association of CAT domains with adaptive coping is partly in line with a study on first-year students [80] that reported poor coping skills to be associated with negative mental health outcomes and female sex to be related to poorer coping.

Sex differences may well be related to neurophysiological sex differences reported in response to early life stressors [81]. For example, emotional abuse was associated with reduced hippocampus volume, specifically in the left hemisphere, in healthy males but not in healthy females [82]. Furthermore, emotional abuse was associated with overall levels of self-reported positive, negative and depressive symptoms with no main or interaction effect of sex. Due to low and high prevalence rates, respectively, sexual abuse and physical neglect had not been explored. The authors [82] concluded that, while females may be more resilient to the neurological effects of CAT, especially emotional abuse, they may be not more resilient to the mental health problems associated with it. As our results indicate, this negative association with mental health might be aggravated by the development of more maladaptive coping strategies in females who had experienced emotional abuse.

### Strengths and limitations

Next to the strengths and limitations that have already been discussed, a clear limitation is the cross-sectional design of our study that, strictly, does not allow to draw causal conclusions. Yet, in our mostly adult sample, the retrospectively reported CAT had occurred explicitly many years earlier in childhood, i.e., “when I was young” [25], and coping strategies were reported as relatively stable over time with only small and unsubstantial changes over 2 years [48]. Thus, coping strategies have likely been well existent before the onset of the state assessment of depression (past two weeks), social phobia (open questions about frequencies) and CHR-P criteria (require an onset or at least qualitative worsening within the past 12 months and an absence in the subjectively defined pre-morbid state, respectively). However, in particular depression and social phobia, might have occurred early in life [49, 50] and influenced the development of coping strategies so that a reciprocal association between these two outcomes and coping strategies cannot be ruled out and might in parts explain the descriptively stronger association of these two outcomes with coping compared to CHR-P.

Moreover, a bias in favor of high associations between self-report measures might have been introduced by common-method variances in our study, as depression and social anxiety as well as CAT and coping were assessed using self-report questionnaires and only CHR-P was assessed using clinical interviews. However, despite some inherent methodological problems [83–85], assessing CAT as well as coping by self-report questionnaires is common-practice and does not per se reflect method variance rather than the intended true construct variance [86]. This is reflected by the fact that the main model remained essentially the same when BDI and SPAI were dichotomized or entered as continuous variable. In particular, the path between maladaptive coping and CHR-P showed the same effect and the explained variance by CHR-P remained the same in both models despite the slightly higher path coefficients and explained variance of BDI and SPAI in the metric model. In addition, the common-practice retrospective assessment of CAT might be prone to recall bias, such as a better recall of negative events in persons with depression [87].

Another limitation to the generalizability of our results is the predominant German nationality of our sample that primarily reflects distributions of CAT domains in Europe [52]. To better understand cultural differences that might shape the relation between CAT and mental health outcome, future studies should include patients of different ethnic backgrounds, in order to be able to compare not only sex but also cultural differences. Furthermore, in future studies, CAT domains should be stratified according to their severity to explore, as expected by McLafferty et al. [80], if moderate levels of certain CAT domains, in particular neglect, are associated with increased resilience. Additionally, using structural equation of latent factors rather than path modelling of observed factors in larger samples might shed light on the differential contributions of the various negative childhood experiences and coping strategies that were summarized in our study as five and two predictors, respectively, as well as of the various signs and symptoms that build up our binary outcome variables.

## Conclusion

Our results support earlier notions of a more dominant mediation role of maladaptive than of adaptive coping strategies in the association between various CAT domains, in particular emotional abuse, and mental health outcomes in patients of an early detection service. Despite earlier reports of CAT being a significant risk factor of psychosis, most direct effects of CAT domains were on both depression and social anxiety rather than on a CHR-P state, which, surprisingly, was unrelated to its two main comorbidities; this, highlighting the need to study the complex interplay of CAT and psychological and biological moderators on mental health in complex models that include relevant interactions to avoid drawing possibly incorrect conclusions from simpler analyses. Such simpler association analyses have repeatedly indicated a strong role for sexual abuse, while our analyses support notions that sexual abuse may mainly add to the overall burden of CAT in those most likely afflicted also by other forms of CAT. Furthermore, the difference between the male and female model support notions of important sex-related differences that call for more integration of psychological, environmental and neurobiological predictors. Finally, in line with the inauguration model of resilience, our results indicated that moderate levels of neglect might be positively associated with factors of resilience, a possibility that also needs further exploration and a more differential view on the primary coping strategies at least on childhood neglect. Such a detailed understanding of the effects of CAT will in future help to develop a multi-dimensional, holistic and sex-specific approach to the treatment of patients who have experienced CAT.

## Supplementary Information

Additional file 1: Comparison of the original and metric path model of the total sample (*N*=736). Comparison of the standardized path coefficients, their 95% confidence intervals and their significance level, and of the explained variance (R^2^) of the mediators and outcomes of the path models with dichotomized and continuous scores of the Beck Depression Inventory (BDI) and Social Phobia and Anxiety Inventory (SPAI).

Additional file 2: Comparison of the original and metric mediation analyses of the total sample (*N*=736). Comparison of the standardized path coefficients and their significance level of the mediation analyses with dichotomized and continuous scores of the Beck Depression Inventory (BDI) and Social Phobia and Anxiety Inventory (SPAI).

Additional file 3: Comparison of the original and metric path model of the male subsample (*n*=490). Comparison of the standardized path coefficients, their 95% confidence intervals and their significance level, and of the explained variance (R^2^) of the mediators and outcomes of the path models with dichotomized and continuous scores of the Beck Depression Inventory (BDI) and Social Phobia and Anxiety Inventory (SPAI).

Additional file 4: Comparison of the original and metric path model of the female subsample (*n*=246). Comparison of the standardized path coefficients, their 95% confidence intervals and their significance level, and of the explained variance (R^2^) of the mediators and outcomes of the path models with dichotomized and continuous scores of the Beck Depression Inventory (BDI) and Social Phobia and Anxiety Inventory (SPAI).

Additional file 5: Comparison of the original and metric mediation analyses of the male subsample (*n*=490). Comparison of the standardized path coefficients and their significance level of the mediation analyses with dichotomized and continuous scores of the Beck Depression Inventory (BDI) and Social Phobia and Anxiety Inventory (SPAI).

Additional file 6: Comparison of the original and metric mediation analyses of the female subsample (*n*=246). Comparison of the standardized path coefficients and their significance level of the mediation analyses with dichotomized and continuous scores of the Beck Depression Inventory (BDI) and Social Phobia and Anxiety Inventory (SPAI).

## Data Availability

The datasets used and/or analyzed during the current study are available from the corresponding author on reasonable request.

## Abbreviations

CAT: Childhood adversities and traumata
CHR-P: Clinical high-risk of psychosis
DSM-IV: Diagnostic and Statistical Manual of Mental Disorders, 4th edition
ICD-10: International Classification of Diseases and Related Health Problems, 10th revision
TADS: Trauma and Distress Scale
SVF-120: German Stress-Coping-Questionnaire
BDI: Beck Depression Inventory
SPAI: Social Phobia and Anxiety Inventory
SIPS: Structured Interview for Psychosis-Risk Syndromes
CFI: Comparative fit index
RMSEA: Root-mean-square error of approximation
SRMR: Standardized root mean square residual
CIs: Confidence intervals
PAF: Population attributable fraction
ISCED: International Standard Classification of Education
SD: Standard deviation
V: Cramer’s V for categorical variables
r: Rosenthal’s r for ordinal variables and non-normally distributed parametric variables
