# Supplementary material for: Do coping strategies mediate the effects of childhood adversities and traumata on clinical high-risk of psychosis, depression, and social phobia? A cross-sectional study on patients of an early detection service
Source: BMC Psychiatry. 2025 Jan 7;25:21. doi: 10.1186/s12888-024-06435-2 (PMC11708078; doi:10.1186/s12888-024-06435-2)
Supplement: Supplementary file 1 — Additional file 1: Comparison of the original and metric path model of the total sample (N=736). Comparison of the standardized path coefficients, their 95% confidence intervals and their significance level, and of the explained variance (R2) of the mediators and outcomes of the path models with dichotomized and continuous scores of the Beck Depression Inventory (BDI) and Social Phobia and Anxiety Inventory (SPAI). [file 12888_2024_6435_MOESM1_ESM.pdf]

Additional file 1: Comparison of the original and metric path model of the total sample (N=736). Comparison of the standardized path coefficients, their 95% confidence intervals and their significance level, and of the explained variance (R^2) of the mediators and outcomes of the path models with dichotomized and continuous scores of the Beck Depression Inventory (BDI) and Social Phobia and Anxiety Inventory (SPAI).

	**Original path model**			**Metric path model**		
$\chi 2$	28.605 (df=15; p=0.018)			80.923 (df=15; p<0.001)		
CFI	0.990			0.961		
RMSEA	0.035 (90%CI=0.014-0.054)			0.077 (90%CI=0.061-0.094)		
SRMR	0.029			0.045		
	**Standardized Beta**	**Lower bound of 95%-CI**	**Upper bound of 95%-CI**	**Standardized Beta**	**Lower bound of 95%-CI**	**Upper bound of 95%-CI**
**Adaptive coping ~**	R^2=0.009			R^2=0.009		
Emotional abuse	-0.113*	-0.201	-0.024	-0.118**	-0.206	-0.030
Emotional neglect	-0.233***	-0.333	-0.133	-0.228***	-0.329	-0.128
Sexual abuse	0.005	-0.082	0.093	0.006	-0.081	0.094
Physical abuse	0.029	-0.063	0.120	0.027	-0.065	0.118
Physical neglect	0.161**	0.061	0.262	0.158**	0.058	0.258
**Maladaptive coping ~**	R^2=0.114			R^2=0.115		
Emotional abuse	0.320***	0.238	0.402	0.329***	0.248	0.410
Emotional neglect	0.084	-0.014	0.181	0.079	-0.018	0.175
Sexual abuse	0.037	-0.047	0.120	0.038	-0.045	0.121
Physical abuse	-0.075	-0.162	0.012	-0.076	-0.162	0.011
Physical neglect	-0.053	-0.149	0.043	-0.048	-0.143	0.048
**Clinical high-risk ~**	R^2=0.004			R^2=0.006		
Adaptive coping	0.039	-0.040	0.119	0.046	-0.033	0.125
Maladaptive coping	0.102*	0.024	0.179	0.101*	0.023	0.178
**Social phobia ~**	R^2=0.256			R^2=0.381		
Adaptive coping	-0.101*	-0.181	-0.021	-0.120**	-0.192	-0.049
Maladaptive coping	0.483***	0.417	0.550	0.587***	0.531	0.643
**Depression ~**	R^2=0.311			R^2=0.387		
Adaptive coping	-0.168***	-0.239	-0.097	-0.239***	-0.301	-0.176
Maladaptive coping	0.452***	0.388	0.517	0.554***	0.500	0.608
**Adaptive coping ~~**						
Maladaptive coping	-0.161***	-0.236	-0.086	-0.163***	-0.237	-0.088
**Clinical high-risk ~~**						
Social phobia	0.050	-0.040	0.141	0.011	-0.080	0.102
Depression	0.004	-0.077	0.086	0.018	-0.064	0.100
**Social phobia ~~**						
Depression	0.120*	0.029	0.210	0.192***	0.101	0.284
**Emotional abuse ~~**						
Emotional neglect	0.432***	0.373	0.491	0.432***	0.373	0.491
Physical abuse	0.386***	0.324	0.447	0.386***	0.324	0.447
Physical neglect	0.376***	0.314	0.438	0.376***	0.314	0.438
Sexual abuse	0.264***	0.197	0.331	0.264***	0.197	0.331

	**Original path model**			**Metric path model**		
	**Standardized Beta**	**Lower bound of 95%-CI**	**Upper bound of 95%-CI**	**Standardized Beta**	**Lower bound of 95%-CI**	**Upper bound of 95%-CI**
**Emotional neglect ~~**						
Physical abuse	0.159***	0.089	0.230	0.159***	0.089	0.230
Physical neglect	0.643***	0.601	0.685	0.643***	0.601	0.685
Sexual abuse	0.178***	0.108	0.248	0.178***	0.108	0.248
**Physical abuse ~~**						
Physical neglect	0.268***	0.201	0.335	0.268***	0.201	0.335
**Sexual abuse ~~**						
Physical abuse	0.522***	0.470	0.575	0.522***	0.470	0.575
Physical neglect	0.256***	0.189	0.324	0.256***	0.189	0.324

* $p \leq 0.05$; ** $p \leq 0.01$; *** $p \leq 0.001$

~ predicted by; ~~ correlated with

$\chi 2$=Chi-Square; df=degrees of freedom; CFI= Comparative Fit Index; RMSEA= Root Mean Square Error of Approximation; SRMR=Standardized Root Mean Square Residual; CI=Confidence Intervall; R^2=Explained variance
