# Supplementary material for: Do coping strategies mediate the effects of childhood adversities and traumata on clinical high-risk of psychosis, depression, and social phobia? A cross-sectional study on patients of an early detection service
Source: BMC Psychiatry. 2025 Jan 7;25:21. doi: 10.1186/s12888-024-06435-2 (PMC11708078; doi:10.1186/s12888-024-06435-2)
Supplement: Supplementary file 3 — Additional file 3: Comparison of the original and metric path model of the male subsample (n=490). Comparison of the standardized path coefficients, their 95% confidence intervals and their significance level, and of the explained variance (R2) of the mediators and outcomes of the path models with dichotomized and continuous scores of the Beck Depression Inventory (BDI) and Social Phobia and Anxiety Inventory (SPAI). [file 12888_2024_6435_MOESM3_ESM.pdf]

Additional file 3: Comparison of the original and metric path model of the male subsample (n=490). Comparison of the standardized path coefficients, their 95% confidence intervals and their significance level, and of the explained variance (R^2) of the mediators and outcomes of the path models with dichotomized and continuous scores of the Beck Depression Inventory (BDI) and Social Phobia and Anxiety Inventory (SPAI).

	**Original path model**			**Metric path model**		
$\chi 2$	23.412 (df=15; p=0.076)			44.335 (df=15; p<0.001)		
CFI	0.991			0.967		
RMSEA	0.035 (90%CI=0.000-0.066)			0.075 (90%CI=0.051-0.101)		
SRMR	0.032			0.038		
	**Standardized Beta**	**Lower bound of 95%-CI**	**Upper bound of 95%-CI**	**Standardized Beta**	**Lower bound of 95%-CI**	**Upper bound of 95%-CI**
**Adaptive coping ~**	R^2=0.094			R^2=0.096		
Emotional abuse	-0.178***	-0.283	-0.074	-0.189***	-0.293	-0.086
Emotional neglect	-0.288***	-0.403	-0.173	-0.282***	-0.397	-0.167
Sexual abuse	-0.000	-0.104	0.103	0.003	-0.100	0.106
Physical abuse	0.034	-0.076	0.144	0.032	-0.077	0.142
Physical neglect	0.201***	0.083	0.319	0.198***	0.080	0.316
**Maladaptive coping ~**	R^2=0.109			R^2=0.116		
Emotional abuse	0.310***	0.211	0.408	0.321***	0.223	0.418
Emotional neglect	0.095	-0.019	0.209	0.091	-0.023	0.204
Sexual abuse	0.004	-0.099	0.107	0.008	-0.094	0.110
Physical abuse	-0.094	-0.201	0.013	-0.096	-0.203	0.010
Physical neglect	-0.012	-0.129	0.105	-0.007	-0.122	0.109
**Clinical high-risk ~**	R^2=0.013			R^2=0.012		
Adaptive coping	0.051	-0.047	0.149	0.056	-0.042	0.154
Maladaptive coping	0.114	0.018	0.210	0.107*	0.011	0.202
**Social phobia ~**	R^2=0.276			R^2=0.394		
Adaptive coping	-0.128**	-0.225	-0.031	-0.146***	-0.235	-0.057
Maladaptive coping	0.480***	0.398	0.562	0.576***	0.506	0.647
**Depression ~**	R^2=0.238			R^2=0.427		
Adaptive coping	-0.165***	-0.255	-0.076	-0.247***	-0.324	-0.169
Maladaptive coping	0.421***	0.338	0.504	0.549***	0.481	0.616
**Adaptive coping ~~**						
Maladaptive coping	-0.174***	-0.265	-0.082	-0.173***	-0.264	-0.082
**Clinical high-risk ~~**						
Social phobia	0.078	-0.030	0.186	0.061	-0.048	0.169
Depression	0.039	-0.060	0.138	0.015	-0.085	0.115
**Social phobia ~~**						
Depression	0.124*	0.015	0.234	0.176**	0.061	0.292
**Emotional abuse ~~**						
Emotional neglect	0.382***	0.306	0.458	0.382***	0.306	0.458
Physical abuse	0.376***	0.300	0.452	0.376***	0.300	0.452
Physical neglect	0.371***	0.295	0.448	0.371***	0.295	0.448
Sexual abuse	0.249***	0.166	0.332	0.249***	0.166	0.332

Addition to “Do coping strategies mediate the effects of childhood adversities and traumata on clinical high-risk of psychosis, depression, and social phobia? A cross-sectional study on patients of an early detection service” by Chang et al.

	**Original path model**			**Metric path model**		
	**Standardized Beta**	**Lower bound of 95%-CI**	**Upper bound of 95%-CI**	**Standardized Beta**	**Lower bound of 95%-CI**	**Upper bound of 95%-CI**
**Emotional neglect ~~**						
Physical abuse	0.099*	0.012	0.187	0.099*	0.012	0.187
Physical neglect	0.616***	0.561	0.671	0.616***	0.561	0.671
Sexual abuse	0.078	-0.010	0.166	0.078	-0.010	0.166
**Physical abuse ~~**						
Physical neglect	0.242***	0.159	0.326	0.242***	0.159	0.326
**Sexual abuse ~~**						
Physical abuse	0.534***	0.471	0.597	0.534***	0.471	0.597
Physical neglect	0.247***	0.164	0.330	0.247***	0.164	0.330

* $p \leq 0.05$; ** $p \leq 0.01$; *** $p \leq 0.001$

~ predicted by; ~~ correlated with

$\chi 2$=Chi-Square; df=degrees of freedom; CFI= Comparative Fit Index; RMSEA= Root Mean Square Error of Approximation; SRMR=Standardized Root Mean Square Residual; CI=Confidence Intervall; R^2=Explained variance
