# Supplementary material for: Do coping strategies mediate the effects of childhood adversities and traumata on clinical high-risk of psychosis, depression, and social phobia? A cross-sectional study on patients of an early detection service
Source: BMC Psychiatry. 2025 Jan 7;25:21. doi: 10.1186/s12888-024-06435-2 (PMC11708078; doi:10.1186/s12888-024-06435-2)
Supplement: Supplementary file 4 — Additional file 4: Comparison of the original and metric path model of the female subsample (n=246). Comparison of the standardized path coefficients, their 95% confidence intervals and their significance level, and of the explained variance (R2) of the mediators and outcomes of the path models with dichotomized and continuous scores of the Beck Depression Inventory (BDI) and Social Phobia and Anxiety Inventory (SPAI). [file 12888_2024_6435_MOESM4_ESM.pdf]

Additional file 4: Comparison of the original and metric path model of the female subsample (n=246). Comparison of the standardized path coefficients, their 95% confidence intervals and their significance level, and of the explained variance (R^2) of the mediators and outcomes of the path models with dichotomized and continuous scores of the Beck Depression Inventory (BDI) and Social Phobia and Anxiety Inventory (SPAI).

	**Original path model**			**Metric path model**		
$\chi 2$	14.835 (df=15; p=0.463)			61.124 (df=15; p<0.001)		
CFI	1.000			0.910		
RMSEA	0.000 (90%CI=0.000-0.072)			0.131 (90%CI=0.096-0.168)		
SRMR	0.031			0.068		
	**Standardized Beta**	**Lower bound of 95%-CI**	**Upper bound of 95%-CI**	**Standardized Beta**	**Lower bound of 95%-CI**	**Upper bound of 95%-CI**
**Adaptive coping ~**	R^2=0.009			R^2=0.009		
Emotional abuse	0.002	-0.159	0.163	0.005	-0.156	0.165
Emotional neglect	-0.134	-0.329	0.061	-0.134	-0.328	0.061
Sexual abuse	0.014	-0.149	0.177	0.013	-0.150	0.176
Physical abuse	-0.012	-0.183	0.159	-0.016	-0.187	0.154
Physical neglect	0.100	-0.085	0.285	0.100	-0.084	0.285
**Maladaptive coping ~**	R^2=0.114			R^2=0.115		
Emotional abuse	0.321***	0.175	0.468	0.324***	0.178	0.469
Emotional neglect	0.036	-0.149	0.220	0.041	-0.142	0.224
Sexual abuse	0.048	-0.101	0.197	0.039	-0.109	0.188
Physical abuse	0.033	-0.125	0.191	0.038	-0.119	0.195
Physical neglect	-0.118	-0.289	0.054	-0.123	-0.293	0.047
**Clinical high-risk ~**	R^2=0.004			R^2=0.006		
Adaptive coping	0.032	-0.103	0.167	0.039	-0.096	0.173
Maladaptive coping	0.060	-0.072	0.192	0.071	-0.060	0.202
**Social phobia ~**	R^2=0.256			R^2=0.381		
Adaptive coping	-0.083	-0.223	0.056	-0.095	-0.219	0.028
Maladaptive coping	0.490***	0.376	0.603	0.599***	0.505	0.692
**Depression ~**	R^2=0.311			R^2=0.387		
Adaptive coping	-0.179**	-0.296	-0.061	-0.227***	-0.336	-0.118
Maladaptive coping	0.508***	0.404	0.612	0.552***	0.458	0.645
**Adaptive coping ~~**						
Maladaptive coping	-0.111	-0.242	0.019	-0.117	-0.246	0.013
**Clinical high-risk ~~**						
Social phobia	0.001	-0.159	0.162	-0.089	-0.250	0.071
Depression	-0.080	-0.223	0.063	0.019	-0.124	0.162
**Social phobia ~~**						
Depression	0.135	-0.027	0.296	0.217**	0.067	0.367
**Emotional abuse ~~**						
Emotional neglect	0.489***	0.394	0.584	0.489***	0.394	0.584
Physical abuse	0.431***	0.329	0.532	0.431***	0.329	0.532
Physical neglect	0.380***	0.273	0.487	0.380***	0.273	0.487
Sexual abuse	0.261***	0.145	0.378	0.261***	0.145	0.378

	**Original path model**			**Metric path model**		
	**Standardized Beta**	**Lower bound of 95%-CI**	**Upper bound of 95%-CI**	**Standardized Beta**	**Lower bound of 95%-CI**	**Upper bound of 95%-CI**
**Emotional neglect ~~**						
Physical abuse	0.280***	0.164	0.395	0.280***	0.164	0.395
Physical neglect	0.687***	0.621	0.753	0.687***	0.621	0.753
Sexual abuse	0.262***	0.146	0.379	0.262***	0.146	0.379
**Physical abuse ~~**						
Physical neglect	0.320***	0.208	0.432	0.320***	0.208	0.432
**Sexual abuse ~~**						
Physical abuse	0.570***	0.486	0.654	0.570***	0.486	0.654
Physical neglect	0.271***	0.155	0.386	0.271***	0.155	0.386

* $p \leq 0.05$; ** $p \leq 0.01$; *** $p \leq 0.001$

~ predicted by; ~~ correlated with

$\chi 2$=Chi-Square; df=degrees of freedom; CFI= Comparative Fit Index; RMSEA= Root Mean Square Error of Approximation; SRMR=Standardized Root Mean Square Residual; CI=Confidence Intervall; R^2=Explained variance
