# Supplementary material for: Do coping strategies mediate the effects of childhood adversities and traumata on clinical high-risk of psychosis, depression, and social phobia? A cross-sectional study on patients of an early detection service
Source: BMC Psychiatry. 2025 Jan 7;25:21. doi: 10.1186/s12888-024-06435-2 (PMC11708078; doi:10.1186/s12888-024-06435-2)
Supplement: Supplementary file 6 — Additional file 6: Comparison of the original and metric mediation analyses of the female subsample (n=246). Comparison of the standardized path coefficients and their significance level of the mediation analyses with dichotomized and continuous scores of the Beck Depression Inventory (BDI) and Social Phobia and Anxiety Inventory (SPAI). [file 12888_2024_6435_MOESM6_ESM.pdf]

Additional file 6: Comparison of the original and metric mediation analyses of the female subsample (n=246). Comparison of the standardized path coefficients and their significance level of the mediation analyses with dichotomized and continuous scores of the Beck Depression Inventory (BDI) and Social Phobia and Anxiety Inventory (SPAI).

			**Original path model**			**Metric path model**		
**Independent variable**	**Mediator variable**	**Dependent variable**	**Direct effect†**	**Indirect effect‡**	**Total effect§**	**Direct effect†**	**Indirect effect‡**	**Total effect§**
emotional abuse	maladaptive coping	depression	0.140*	0.160***	0.300***	0.300***	0.153***	0.453***
emotional abuse	maladaptive coping	social phobia	0.035	0.154***	0.189**	0.137*	0.178***	0.315***

* p≤0.05; ** p≤0.01; *** p≤0.001

† Direct effect=Effect of independent variable on dependent variable (c)

‡ Indirect effect=Product of effects of independent variable on mediator variable (a) and mediator variable on dependent variable (b)

§ Total effect=c+(a*b)
